# FKBP51: A new target for Parkinson’s disease

**DOI:** 10.4103/NRR.NRR-D-25-00651

**Published:** 2025-09-29

**Authors:** Marta Garcia-Gomara, Mar Cuadrado-Tejedor, Ana Garcia-Osta

**Affiliations:** Gene Therapy for CNS Disorders program, Center for Applied Medical Research (CIMA), University of Navarra, Pamplona, Spain; IdiSNA (Navarra Institute for Health Research), Pamplona, Spain; Department of Pathology, Anatomy and Physiology, School of Medicine, University of Navarra, Pamplona, Spain

Parkinson’s disease (PD) is a progressive age-related neurodegenerative disorder clinically defined by motor symptoms and pathologically by the loss of dopaminergic (DA) neurons in the substantia nigra pars compacta. These neurons are characterized by the presence of the cytoplasmic pigment neuromelanin (NM), and their degeneration is closely associated with the accumulation of α-synuclein (α-syn) into intraneuronal inclusions known as Lewy bodies (LBs), which represent a neuropathological hallmark of PD.

Multiple lines of evidence support a pathological interaction between NM and α-syn in PD. α-Syn has been identified as a major component of NM granules isolated from PD brains, but not from healthy controls (Fasano et al., 2003). LBs are often found forming within or near NM-rich regions (Fasano et al., 2003). Additionally, the age-related accumulation of NM has been proposed to promote α-syn overexpression and aggregation, facilitating LB formation (Xuan et al., 2011). These findings support the hypothesis that NM not only marks vulnerable DA neurons, but may also actively contribute to PD pathogenesis.

To investigate this hypothesis, in our study (Garcia-Gomara et al., 2025), a humanized PD mouse model (Snca^–/–^; PAC-Tg(SNCAWT)) in which NM accumulation is induced in the substantia nigra via stereotactic delivery of human tyrosinase (h-Tyr) was developed. This model integrates three key pathological features of PD: (i) NM accumulation, (ii) human wild-type α-syn overexpression, and (iii) aging. Remarkably, these mice recapitulate progressive motor impairment and DA neurodegeneration with age (Garcia-Gómara et al., 2025).

Upon h-Tyr-induced NM buildup, DA neurons develop p62- and ubiquitin-positive inclusions, reminiscent of early-stage LB pathology. Using transcriptomic profiling, we identified a robust microglial activation signature in the midbrain of NM-SNCAWT mice, indicating that neuroinflammation plays a major role in the degenerative process. Among the most significantly dysregulated genes, *Fkbp5*, which encodes the stress-responsive co-chaperone FKBP51 involved in protein homeostasis and inflammation, was identified. Interestingly, FKBP51 expression was markedly elevated in both aged NM-SNCAWT mice and in human PD midbrains, a finding further supported by analysis of publicly available human transcriptomic datasets.

Targeting this pathway, we tested SAFit2, a highly selective FKBP51 inhibitor. Treatment with SAFit2 reduced the burden of ubiquitin-positive inclusions, preserved DA neurons in the substantia nigra pars compacta, and significantly improved motor performance in the mouse model. These results establish FKBP51 as a key mediator of α-syn/NM-induced neurotoxicity and support SAFit2 as a promising disease-modifying therapeutic candidate for PD.

**Available therapeutic strategies in PD:** There is currently no cure for PD; however, a range of therapeutic interventions can provide symptomatic relief. The gold standard clinical treatment for PD involves restoring dopamine levels in the striatum through the administration of L-DOPA. However, after a prolonged period of use, which can range from 2 to 7 years, this therapy fails to sustain motor symptom control and may, instead, contribute to the development of drug-induced dyskinesia. In addition to pharmacological therapy, other strategies such as deep-brain stimulation and, more recently, high-intensity focused ultrasound have been employed to manage the symptoms of the disease. However, not all patients meet the necessary clinical criteria to be eligible for these interventions. In 2023, there were 136 registered clinical trials for both disease-modifying and symptomatic therapies, but none have yet shown a disease-modifying effect in clinical trials (McFarthing et al., 2024). This indicates that further research is needed in the development of new therapeutic strategies for PD.

**FKBP51 as a promising therapeutic target for PD:** Given the urgent need for novel disease-modifying approaches, identifying new molecular targets is essential. In this context, our study presents a newly developed animal model of PD, which has allowed us to uncover, for the first time, the dysregulation of *Fkbp5*, the gene encoding FK506-binding protein 51 (FKBP51), in the context of PD (Garcia-Gomara et al., 2025). *Fkbp5* was identified as a significantly differentially expressed gene in a bulk RNA-seq analysis of the midbrain from our PD mouse model. This initial finding led us to examine its relevance in human pathology, where we confirmed elevated FKBP51 protein levels by western blot assay and immunofluorescence in postmortem substantia nigra samples from PD patients. Additionally, analysis of publicly available transcriptomic datasets supported the upregulation of FKBP51 in PD-affected regions, underscoring its potential relevance as a novel therapeutic target in PD.

FKBP51 belongs to the FKBP (FK506-binding protein) family, which is part of the broader class of immunophilins—a subgroup of molecular chaperones. High molecular weight immunophilins, such as FKBP51, interact with the 90-kDa heat shock protein (Hsp90), and through modulation of this interaction, they can influence the aggregation or clearance of aberrant proteins—processes that are key pathological features of various neurodegenerative diseases (Zgajnar et al., 2019). In addition, they form a complex with the glucocorticoid receptor that is enhanced under stress-related conditions (Jiang et al., 2022). These evidences position FKBP51 as a critical molecular player across both psychiatric and neurodegenerative disorders.

In this line, genetic variants in the *Fkbp5* gene have been repeatedly linked to psychiatric conditions, particularly those triggered by post-traumatic stress. Elevated FKBP51 expression has also been reported in patients with schizophrenia, further supporting its involvement in stress-related pathophysiology (Matosin et al., 2023). Beyond psychiatry, the role of FKBP51 is now gaining attention in the context of neurodegeneration. Both genetic knockdown and pharmacological inhibition of FKBP5 have led to significant reductions in mutant huntingtin protein levels in mouse models of Huntington’s disease (Bailus et al., 2021). Similarly, suppression of *Fkbp5* via antisense oligonucleotides has been shown to decrease tau accumulation *in vitro* (Gebru et al., 2023), an effect also observed *in vivo* in *Fkbp5*^*⁻/⁻*^ mice. Notably, overexpression of Fkbp51 in a tauopathy mouse model preserved pathogenic tau species, further implicating this co-chaperone in the molecular mechanisms driving AD (Blair et al., 2013). Collectively, these findings suggest that FKBP51 is not merely a passive marker, but an active contributor to neurodegenerative pathology, and, as such, warrants serious consideration as a therapeutic target.

**Figure 1:**
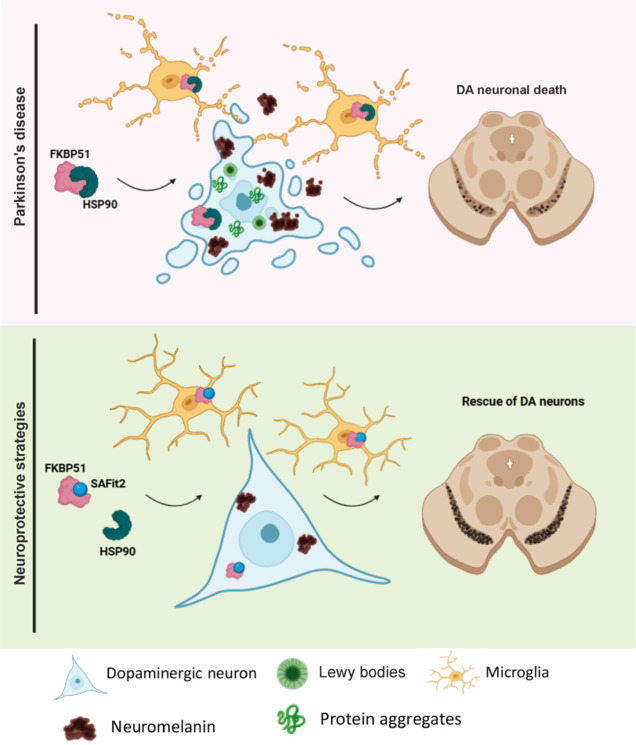
FKBP51 inhibition in the NM mouse model of PD. Pharmacological treatment with SAFit2, a potent FKBP51 inhibitor, resulted in reduced neuroinflammation and LB-like inclusions, thereby preventing neurodegeneration in the substantia nigra in the NM mouse model of PD. These findings underscore the critical role of FKBP51 in PD and support SAFit2 as a promising therapeutic candidate for mitigating neurodegeneration in PD. Created with BioRender.com. DA: Dopaminergic; HSP90: 90-kDa heat shock protein; LB: Lewy body; NM: neuromelanin; PD: Parkinson’s disease.

In our study, we observed a significant upregulation of Fkbp51 in both DA neurons and microglial cells of aged SNCAWT mice, as well as in our novel NM-mouse model of PD induced by the h-Tyr overexpression. Notably, elevated FKBP51 levels were also detected in neurons and microglial cells from post-mortem brain tissue of PD patients compared to healthy individuals (sample size and source details are available at the paper by Garcia-Gomara et al. (2025). These findings align with previous reports showing that FKBP51 expression increases with age in the human brain and is further elevated in AD (Matosin et al., 2023). Given that FKBP51 it is known to synergize with Hsp90 to inhibit tau degradation and promote pathogenic tau accumulation (Blair et al., 2013), we hypothesized that a similar mechanism may contribute to neurodegeneration in our PD mouse model. In line with this, we demonstrate an increase in FKBP51 and Hsp90 co-expression in DA neurons of the midbrain upon h-Tyr expression, with both proteins forming a chaperone complex, as detected by western blot assay and *in situ* proximity ligation assay. We hypothesize that, similar to what has been described in tauopathy models (Blair et al., 2013), this co-chaperone complex may impair the clearance of misfolded proteins, potentially contributing to progressive degeneration of DA neurons. Consistent with this hypothesis, our results demonstrate that inhibiting FKBP51 with the selective compound SAFit2 prevents the formation of the FKBP51-Hsp90 co-chaperone complex and may modulate HSP90 activity, triggering degradation pathways such as ubiquitination of α-syn. This correlates with reduced ubiquitin- and α-syn-positive inclusions in NM-containing neurons (**Figure 1**), suggesting enhanced autophagic clearance that ultimately mitigates neurodegeneration in a PD mouse model (García-Gomara et al., 2025). While these findings support a mechanism similar to that previously described for Huntington (Bailus et al., 2021) or AD (Jeanne et al., 2024), the direct mechanistic link between FKBP51 inhibition and reduced α-syn accumulation remains to be experimentally confirmed. Clarifying this relationship will be a key objective for future studies. Additional studies support our findings, as other members of the FKBP family, such as FKBP12 and FKBP52, have been shown to accelerate both α-syn aggregation and cell death *in vitro*, while their inhibition reduces these pathological effects (Caminati et al., 2019). These observations further validate FKBPs as novel drug targets for the causative treatment of PD.

In addition to protein aggregation, chronic neuroinflammation has emerged as a central driver of neurodegeneration in PD, with microglia playing a pivotal role. A key pathological mechanism involves the release of NM granules from dying DA neurons, which in turn activate surrounding microglia. This extracellular NM acts as a potent immunogenic signal, inducing a reactive microglial state that amplifies neuroinflammatory responses and accelerates the degeneration of neighboring neurons (Garcia-Gomara et al., 2025). Our study also identified microglia as a cell population exhibiting significant upregulation of FKBP51 in brain samples of PD patients. This observation is further substantiated by transcriptomic analyses showing elevated *FKBP5* mRNA levels in microglia from AD brains (Grubman et al., 2019), indicating a conserved role for FKBP51 across neurodegenerative contexts. Importantly, pharmacological inhibition of FKBP51 using SAFit2 reduced its expression in microglia and mitigated downstream inflammatory responses (**Figure 1**). These results suggest that FKBP51 not only contributes to neuronal vulnerability but also actively participates in the neuroimmune crosstalk that perpetuates degeneration. Targeting FKBP51 in microglia may therefore represent a promising dual-action therapeutic strategy—addressing both proteinopathy and inflammation in PD.

**Challenges and future directions:** Despite growing evidence supporting FKBP51 as a promising therapeutic target in neurodegenerative diseases, several important challenges remain. The multifunctional nature of FKBP51, along with its differential expression across cell types and disease contexts, requires a deeper understanding of its mechanistic roles. In PD, for instance, FKBP51 is upregulated in both DA neurons and microglial cells. Elucidating its specific functions in these distinct cellular populations is essential for the development of more precise and effective cell-specific therapeutic strategies. Moreover, while pharmacological inhibitors such as SAFit2 have shown considerable promise in preclinical studies, the efficacy and safety of FKBP51-targeted therapies must be thoroughly validated before clinical application. In particular, long-term toxicity studies are needed to evaluate the potential consequences of chronic FKBP51 inhibition in the context of PD treatment. Looking ahead, the integration of molecular profiling, cell type–specific modulation, and innovative delivery platforms will be critical to advancing FKBP51-based interventions from bench to bedside.

Ongoing research is increasingly focused on strategies that not only inhibit protein function but also promote targeted protein degradation. One such approach involves the use of Proteolysis Targeting Chimeras—bifunctional molecules designed to simultaneously bind a specific target protein and recruit an E3 ubiquitin ligase, thereby directing the target for ubiquitination and subsequent proteasomal degradation. In 2024, the SelDeg51 Proteolysis Targeting Chimeras was shown to induce efficient degradation of FKBP51 via the proteasome, effectively eliminating the protein from the cell (Geiger et al., 2024). In contrast, selective inhibitors such as SAFit2 block FKBP51 activity while leaving the protein intact. Proteolysis Targeting Chimeras technology offers the potential for more complete and sustained suppression of proteins like FKBP51. Nonetheless, Geiger et al. (2024) reported that the degradation efficiency of FKBP51 could still be improved through further molecular optimization, highlighting the need for continued refinement of this promising therapeutic strategy.

**Conclusion and final remarks:** While current treatments for PD offer symptomatic relief, they do not address the underlying neurodegenerative processes, underscoring the urgent need for disease-modifying therapies with true neuroprotective potential. A critical step toward this goal is the use of animal models that more faithfully recapitulate the complex neuropathology observed in patients. In this regard, the NM-SNCA mouse model provides a valuable platform for investigating disease mechanisms and testing novel therapeutic approaches. Within this framework, our study highlights FKBP51 as a particularly promising target. The protective effects observed following its inhibition suggest that FKBP51 actively contributes to disease progression. Thus, modulating FKBP51 activity may offer a new therapeutic strategy — not only for symptom management, but also for slowing or altering the course of PD.

*This work was supported by PID2022-138285OB-I00, financiado por MCIN/AEI/10.13039/501100011033/FEDER, UE to AGO and MCT, and by Asociación de Amigos fellowship grant to MGG.*
